# Human umbilical cord mesenchymal stem cells reduce oxidative damage and apoptosis in diabetic nephropathy by activating Nrf2

**DOI:** 10.1186/s13287-021-02447-x

**Published:** 2021-08-11

**Authors:** Ping Nie, Xue Bai, Yan Lou, Yuexin Zhu, Shan Jiang, Lina Zhang, Na Tian, Ping Luo, Bing Li

**Affiliations:** 1 Department of Nephropathy, The Second Hospital of Jilin University, Changchun, Jilin Province China; 2 Research and Development Department, Jilin Tuohua Biotechnology Co., Ltd., Changchun, Jilin Province China

**Keywords:** Mesenchymal stem cell, Oxidative damage, Apoptosis, Diabetic nephropathy, Nrf2

## Abstract

**Background:**

Mesenchymal stem cells (MSCs) have a therapeutic effect on diabetic nephropathy (DN) but the underlying mechanism remains unclear. This study was conducted to investigate whether human umbilical cord-MSCs (hUCMSCs) can induce oxidative damage and apoptosis by activating Nrf2.

**Methods:**

We used a type 2 diabetic rat model and a high-glucose and fat-stimulated human glomerular mesangial cell (hGMC) model. Western blotting, RT-qPCR, and TUNEL staining were performed on animal tissues and cultured cells. Nuclear expression of Nrf2 was detected in the renal tissue. Furthermore, Nrf2 siRNA was used to examine the effects of hUCMSCs on hGMCs. Finally, the effect of hUCMSCs on the Nrf2 upstream signalling pathway was investigated.

**Results:**

After treatment with hUCMSCs, Nrf2 showed increased expression and nuclear translocation. After Nrf2-specific knockout in hGMCs, the protective effect of hUCMSCs on apoptosis induced by high-glucose and fat conditions was reduced. Activation of the PI3K signalling pathway may be helpful for ameliorating DN using hUCMSCs.

**Conclusions:**

hUCMSCs attenuated renal oxidative damage and apoptosis in type 2 diabetes mellitus and Nrf2 activation is one of the important mechanisms of this effect. hUCMSCs show potential as drug targets for DN.

**Supplementary Information:**

The online version contains supplementary material available at 10.1186/s13287-021-02447-x.

## Background

Diabetic nephropathy (DN) is a common but serious complication of type 1 and type 2 diabetes and progressively leads to chronic loss of kidney function and eventual death [1–3]. To date, treatments that can delay the progression of DN are unavailable. Mesenchymal stem cells (MSCs) are a type of undifferentiated cells with multi-differentiation potential, self-renewal ability, and low immunogenicity. Introduction of MSCs is considered a promising method for inducing tissue regeneration in DN [4]. Previous studies showed that MSCs protect against the progression of DN in rats and mice [5–8].

Nuclear factor erythroid 2-related factor 2 (Nrf2) is an important transcription factor that regulates oxidative stress, a condition important in the development of diabetes. Nrf2 not only regulates oxidative damage in type 2 diabetes mellitus [9, 10], but also has an anti-apoptotic effect [11–13]. In diabetes, Nrf2 is activated via the PI3K/Akt signalling pathway, which greatly reduces the level of high glucose-induced cardiomyocyte apoptosis [13]. Nrf2 is also activated via the β-catenin signalling pathway and is involved in preventing apoptosis in renal tubular epithelial cells [12].

A previous study showed that MSCs can reduce lung injury and apoptosis induced by isopropyl alcohol by increasing the expression of Nrf2 [14]. The Nrf2 signalling pathway is activated by bone marrow MSCs and exerts an anti-apoptotic effect in spinal cord injury [15]. Moreover, MSC transplantation has been shown to increase Nrf2 gene expression in the injured livers of mice [16]. Considering that MSCs have been shown to be effective in several diseases in which Nrf2 plays a central role, we predicted that MSCs regulate oxidative damage in DN by affecting Nrf2 expression and the Nrf2 signalling pathway.

In this study, we used a high-fat diet and streptozocin-induced rat model of type 2 diabetes to investigate the role of human umbilical cord-MSCs (hUCMSCs) in inhibiting diabetes-related apoptosis by regulating oxidative stress. In addition, we treated human glomerular mesangial cells (hGMCs) with glucose and palmitate in vitro to examine the protective effect of hUCMSCs on mesangial cells. We further investigated the key role of Nrf2 in hUCMSC-based treatment of DN.

## Methods

### Isolation, culture, and characterization of MSCs

Clinical-grade hUCMSCs were supplied by Jilin Tuohua Biological Co., Ltd. (Siping, China). hUCMSCs were prepared from fresh healthy umbilical cords and washed with phosphate-buffered saline (PBS); the blood vessels were removed and the tissue was peeled from the Fahrenheit glue, cut to 1 mm in size, and cultured in complete medium for mesenchymal stem cells (Tuohua Biological) at 37 °C in a 5% CO_2_ incubator. When the cells grew to approximately 90% confluence, they were digested with 0.25% trypsin and passaged at a ratio of 1:3. The cells were successfully induced to differentiate into adipocytes (Fig. S1a), osteoblasts (Fig. S1b), and chondrocytes (Fig. S1c). Flow cytometry showed that the cells expressed CD73, CD90, and CD105 but did not express CD14, CD19, CD34, CD45, and CD31, nor did they express HLA-DR (Fig. S1d), which met the international identification standard. The cell product was certified by the National Institutes for Food and Drug Control (Report number: SH201301098, SH201301175, SH201301317, SH201500350, SH201500351, SH201500477, SH201701982, SH201701983). hUCMSCs were identified by Tuohua Biological Co., Ltd. using the specific data and pictures provided. Fifth-passage cells were used for subsequent experiments.

### Animal studies

**Fig. 1:**
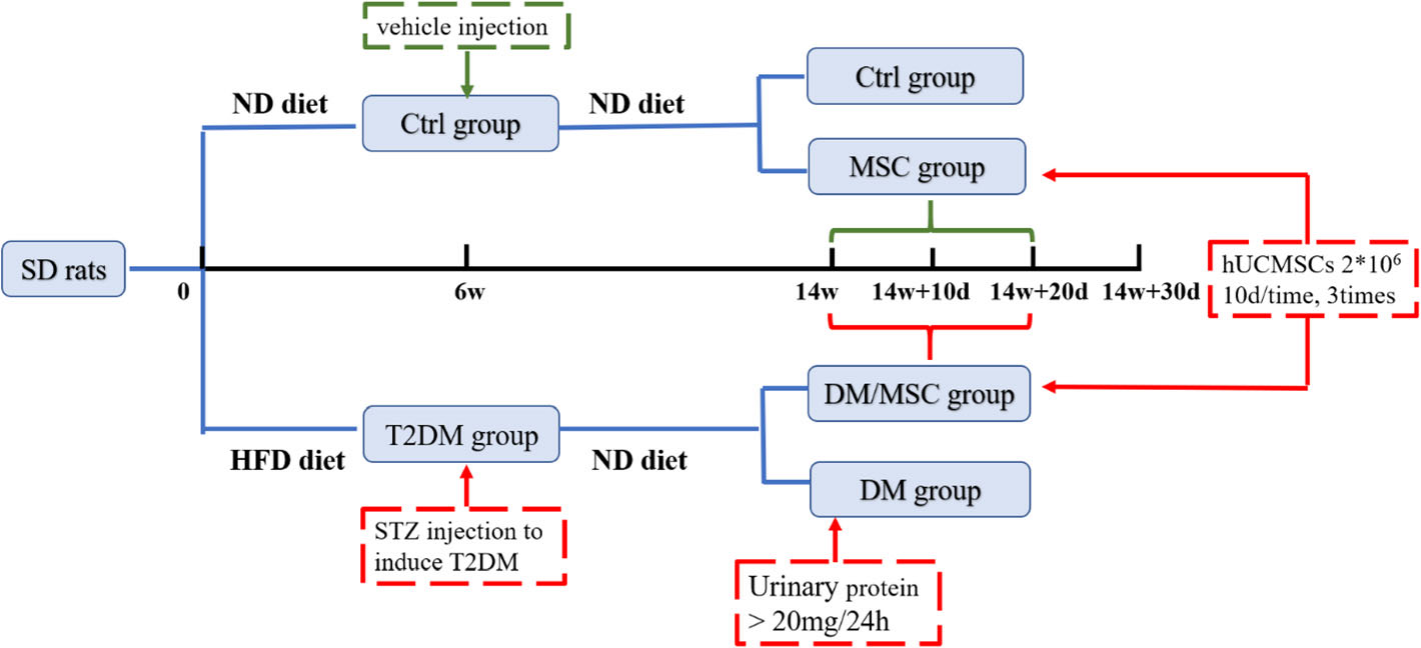
Timeline of animal experiment. *Ctrl*, control rats; *MSC*, control rats injected with hUCMSCs; *DM*, diabetic rats; *DM/MSC*, diabetic rats injected with hUCMSCs; *T2DM*, type 2 diabetes mellitus

The animal experiments were approved by the experimental animal ethics committee of Jilin University (2018SY1101). Seven-week-old male Sprague-Dawley rats were purchased from Changsheng Biotechnology (Liaoning, China) and housed in the Animal Centre of Jilin University. The laboratory temperature was 22 °C and alternating 12-h light/dark cycles were applied. The rats had free access to food and water and moved freely in their cages. Rats in the diabetic group were fed a high-fat diet (Research Diets, New Brunswick, NJ, USA; D12451 rodent diet with 45 kcal% fat) for 6 weeks, whereas control rats were fed a normal diet (ND; Research Diets, D12450B, rodent diet with 10 kcal% fat). Rats in the diabetic group were intraperitoneally injected with streptozotocin (35 mg/kg; Sigma-Aldrich, St. Louis, MO, USA) dissolved in 0.1 M sodium citrate (pH 4.5), and control rats were injected with 0.1 M sodium citrate [17]. Three days after injection of streptozotocin, blood glucose levels were measured in blood drawn through the tail vein for three consecutive days, and a blood glucose level above 250 mg/dL was considered to indicate diabetes [12]. We monitored the body weight and urinary protein levels in the rats every week. After 8 weeks of streptozotocin injection, the urine volume of diabetic rats increased significantly, and the urine protein level was more than 20 mg/24 h. Rats in the ND-fed group were randomly divided into two groups: control rats (Ctrl) and control rats injected with hUCMSCs (MSC). Rats in the high-fat diet-fed group were randomly divided into diabetic rats (DM) and diabetic rats injected with hUCMSCs (DM/MSC), with more than five rats in each group. DM/MSC and MSC rats were injected with fifth-passage hUCMSCs (2 × 10^6^ cells, 1 mL) via the tail vein three times every 10 days [18]. The Ctrl and DM groups were treated with PBS (1 mL) (Fig. 1).

### Cell culture and treatment

hGMCs (FuHeng Bio-technology, Shanghai, China) were cultured in Dulbecco’s modified Eagle’s medium (HyClone, Logan, UT, USA; 5.5 mM glucose) containing 10% foetal bovine serum. To simulate diabetes in vitro, we cultured hGMCs in media containing d-glucose at a final concentration of 30 mM for 24 h and added palmitate (300 mM) in the last 6 h [12]. hUCMSCs were placed in the upper layer of the transwell chamber at a density of 4 × 10^5^ cells /ml and hGMCs were placed in the lower layer of the chamber for 24 h. As the vehicle control, Dulbecco’s modified Eagle’s medium from hGMCs alone was added. The groups were as follows: control group (control), control and hUCMSC group (C/MSC), high-glucose and palmitate group (HG/P), and high-glucose, palmitate, and hUCMSC group (HG/P/MSC).

### Biochemical examination

Blood glucose levels were measured using Accu-Chek test strips (Roche, Basel, Switzerland). Serum lipid, creatinine, and renal malondialdehyde levels were measured using a commercially available kit (Nanjing Jiancheng Bioengineering Institute, Nanjing, China). We measured urinary albumin levels in rats using an automatic chemical analyser (Dirui, Changchun, China) and calculated the urinary albumin/creatinine ratio. The right kidney hypertrophy index was calculated as the ratio of the weight of the right kidney to the total weight of the rat. Glutathione peroxidase (GPX) of cells was measured using Human GPX ELISA Kit (Mlbio, Shanghai, China).

### Renal histopathology assessment

Renal tissues were fixed in 10% formalin for 24 h, embedded in paraffin, sectioned at 3 μm thickness, stained with periodic acid–Schiff stain (Sigma-Aldrich), and observed using a microscope (Olympus, Tokyo, Japan). To prepare ultrathin sections, 1 mm^3^ of renal tissue was fixed in 2.5% glutaraldehyde (pH 7.4) at 4 °C for 24 h. Next, the tissue was fixed with 1% osmium tetroxide for 2–3 h, dehydrated with acetone and ethanol, and embedded with epoxy resin. Slices were prepared using an ultrathin slicing machine at 50 nm thickness and then stained with 3% uranium acetate and lead citrate. The sections were observed under a transmission electron microscope (JEOL, Tokyo, Japan).

### Immunohistochemical staining

Paraffin-embedded kidney tissue sections were incubated with primary anti-Nrf2 antibody (1:100; Abcam, Cambridge, UK) for 16–18 h at 4 °C. The sections were washed with PBS containing 0.1% Tween 20 and incubated with a secondary goat anti-rabbit antibody (1:200; Bioss, Beijing, China) for 1 h at 25 °C. After washing with PBS containing 0.1% Tween 20, the sections were stained with diaminobenzidine.

### TUNEL assay

Paraffin sections were dewaxed, hydrated, and treated with proteinase K without DNase for 30 min at 37 °C. The tissues were then covered with 0.5% Triton X-100 for 15 min. The level of apoptosis was measured with a One-Step TUNEL Cell Apoptosis Detection Kit (Beyotime Biotechnology). The nuclei were counterstained with DAPI (Beyotime Biotechnology), and images were acquired using a fluorescence microscope (Olympus, Tokyo, Japan).

### Isolation of nuclear and cytoplasmic proteins from renal tissue

Nuclear and cytoplasmic proteins from renal tissues were extracted using a nuclear protein extraction kit (Bestbio, Beijing, China). Renal tissue (20 mg) from each rat was placed in 300 μL of extract buffer A containing 1 μL protease and phosphatase inhibitors. After incubation at 4 °C with agitation for 30 min and centrifugation at 2000×*g* for 5 min, the supernatant was used as the cytoplasmic protein. The precipitate was washed twice with PBS, and then 100 μL of cold extract buffer B, containing 0.5 μL protease inhibitor and phosphatase inhibitor, was added. The samples were vigorously mixed at a high speed for 15 s, and the mixture was incubated at 4 °C with agitation for 30 min and subsequently centrifuged at 12,000×*g* for 10 min. The supernatant contained the nucleoproteins.

### Western blotting

Renal tissue was treated with lysis solution (Beyotime Biotechnology, Shanghai, China) and ultrasonicated. Total protein was separated by SDS-PAGE and transferred onto 0.45-μm polyvinylidene difluoride membranes (Millipore, Billerica, MA, USA). After blocking in Tris-buffered saline containing 5% non-fat milk for 1 h at 25 °C, the membranes were incubated for 16–18 h at 4 °C with the following antibodies: anti-4-hydroxynonenal (4-HNE; 1:1000; Abcam), anti-caspase3 (1:1000; Cell Signalling Technology, Danvers, MA, USA), anti-Bcl-2 (1:1000; Abcam), anti-Bax, anti-catalase (CAT, 1:500; Sangon Biotech, Shanghai, China), anti-Nrf2 (1:1000; Abcam), anti-NQO1 (1:8000; Abcam), anti-heme oxygenase 1 (HO-1; 1:1000; Abcam), anti-SOD2 (1:500; Sangon Biotech), anti-Akt, anti-phospho-Akt, anti-PI3K, anti-phospho-PI3K, and β-actin (1:2000, Cell Signalling Technology). The membranes were subsequently incubated with the secondary antibody for 1 h at 25 °C, and protein bands were detected by electrochemiluminescence (Millipore). ImageJ software (NIH, Bethesda, MD, USA) was used to analyse the protein band intensities.

### Real-time qPCR

Total RNA was extracted from renal tissues using an RNA Total Extraction Kit (Promega, Madison, WI, USA) and reverse-transcribed to cDNA using All-in-One RT Master Mix (Transgen Biotech, Beijing, China). Real-time qPCR was performed using an ABI 7300 Real-Time qPCR system (Applied Biosystems, Foster City, CA, USA) using SYBR Green qPCR Master Mix (Transgen Biotech). The primers were synthesised at Sangon Biotech. The primer sequences for Nrf2 and HO-1 were 5′-TAGATCTTGGGGTAAGTCGAGA-3′ and 5′-CTCTTGTCTCTCCTTTTCGAGT-3′, and 5′-TATCGTGCTCGCATGAACACTCTG-3′ and 5′-GTTGAGCAGGAAGGCGGTCTTAG-3′, respectively.

### Immunofluorescence studies

hGMCs were cultured and treated as described in the previous section. Thereafter, the cells were fixed with paraformaldehyde for 15 min, permeabilised with 0.2% Triton X-100 for 10 min, blocked with sheep serum for 30 min, and incubated with anti-Nrf2 antibody (1:200 dilution; Abcam) for 16–18 h at 4 °C, followed by incubation with Alexa Fluor 488-labelled goat anti-rabbit IgG (H+L) antibody (Beyotime Biotechnology) and counterstained with DAPI. Fluorescent staining of the cells was analysed with a confocal microscope.

### Nrf2 siRNA transfection

hGMCs were transfected with human Nrf2 antisense siRNA (NFE2L2-homo-1845; GenePharma, Shanghai, China), and negative controls included cells transfected with sense siRNA (GenePharma) using Lipofectamine™ 2000 (Invitrogen, Carlsbad, CA, USA) transfection reagent for 24 h. Transfections were repeated in high-sugar, high-fat-treated hUCMSCs. The sequence of the sense siRNA was 5′-GCCUGUAAGUCCUGGUCAUTT-3′ and that of the antisense siRNA was 5′-AUGACCAGGACUUACAGGCTT-3′.

### Statistical analysis

Data are presented as the mean ± SD. Comparisons between groups were performed by one-way analysis of variance. The data were graphed using GraphPad Prism 7.0 software (GraphPad Software, Inc., San Diego, CA, USA). Statistical significance was set at *P* < 0.05.

## Results

### Effects of hUCMSCs, and biochemical and physiological indices in type 2 diabetic rats

**Fig. 2:**
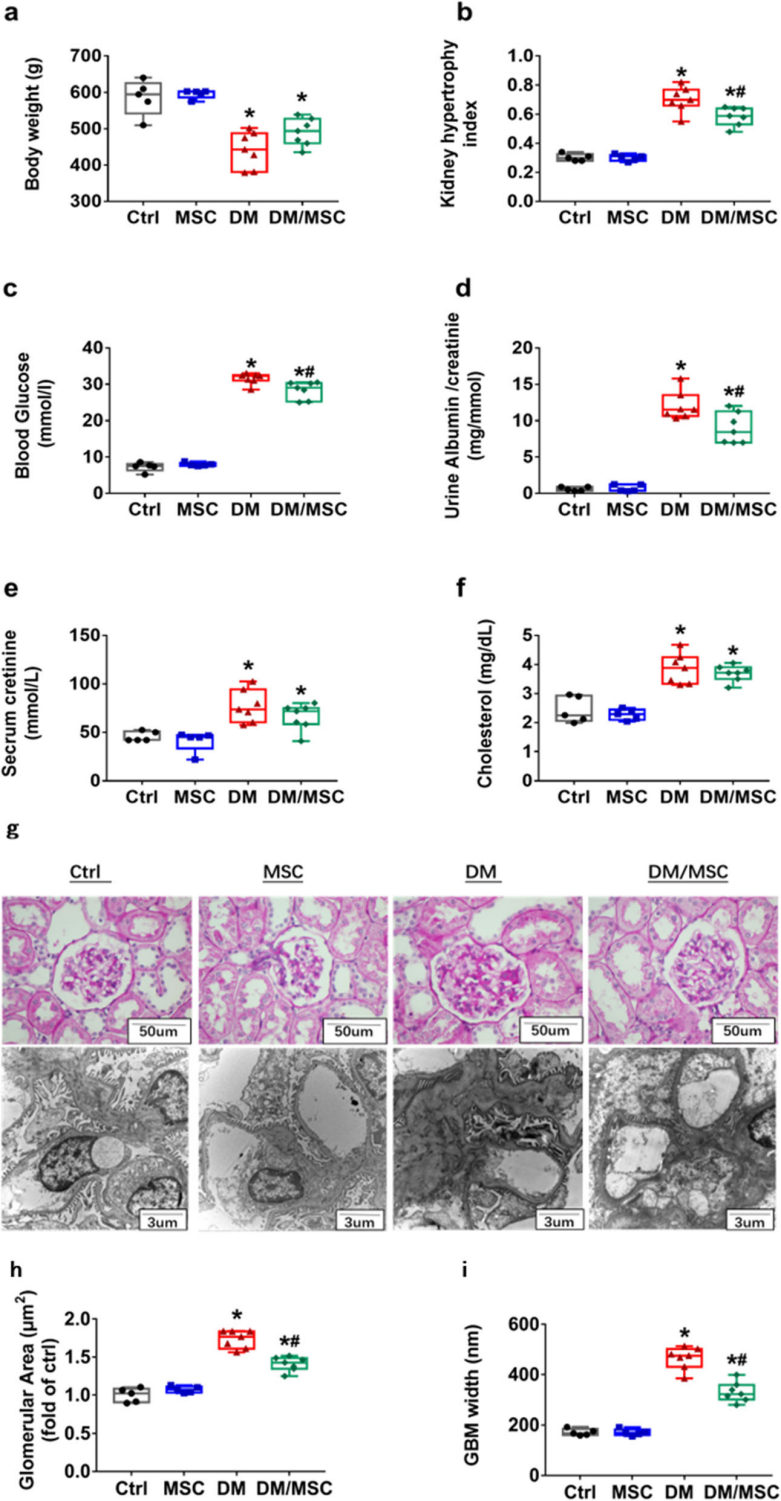
Renoprotective role of human umbilical cord mesenchymal stem cells (hUCMSCs) assessed using general parameters and pathological changes. **a** Body weight. **b** Right kidney hypertrophy index. **c** Blood glucose. **d** Urinary albumin/creatinine ratio. **e** Serum creatinine. **f** Serum cholesterol. **g** Representative images of periodic acid–Schiff staining of kidney tissues from different groups (scale bar = 50 μm) and electron microscopy (scale bar = 3 μm). **h** Glomerular area in different groups. **i** Glomerular basement membrane width. *n* = 5–7 per group. *P < 0.05 vs. Ctrl group; ^*#*^*P <* 0.05 vs DM group

To evaluate the protective effect of hUCMSCs on diabetic rats, we measured the body weight, kidney hypertrophy index, blood glucose, urinary protein, creatinine, and blood lipid levels in diabetic rats. The body weight (Fig. 2a) and kidney hypertrophy index (Fig. 2b) of diabetic rats (DM) were significantly higher than those of the control groups (Ctrl) (*P <* 0.05). Injection of hUCMSCs did not significantly affect the body weight of MSC group rats but significantly reduced the kidney hypertrophy index of diabetic DM/MSC rats (P *<* 0.05). Blood glucose levels were significantly elevated in all diabetic rats (DM) compared to control rats (Ctrl). However, after treatment with hUCMSCs, blood glucose levels were significantly decreased in the DM/MSC group (Fig. 2c, *P* < 0.05). The urinary albumin/creatinine ratio (Fig. 2d) of diabetic rats was significantly higher than that of the control group (*P <* 0.05). After treatment with hUCMSCs, the urinary albumin/creatinine ratio of the DM/MSC group was significantly lower than that of the DM group (*P <* 0.05). Compared with the Ctrl groups, the serum creatinine (Fig. 2e) and cholesterol (Fig. 2f) levels in DM and DM/MSC rats were significantly increased (*P <* 0.05).

The kidney tissues of rats were examined by light microscopy (600×) and electron microscopy (10,000×) (Fig. 2g). Compared with those in the control groups, pathological changes in the kidneys of diabetic rats were obvious and mainly manifested as increases in the glomerular volume, mesangial cell proliferation, and extracellular matrix as well as glomerular basement membrane thickening and epithelial foot process fusion. After hUCMSC treatment, these pathological changes were abrogated, the glomerular area was significantly smaller than that of diabetic rats (Fig. 2h, *P* < 0.05), and epithelial foot process fusion and basement membrane thickening were decreased compared with those in diabetic rats (Fig. 2i, *P* < 0.05).

### Effects of hUCMSCs on diabetes-induced renal oxidative stress and renal cell apoptosis

**Fig. 3:**
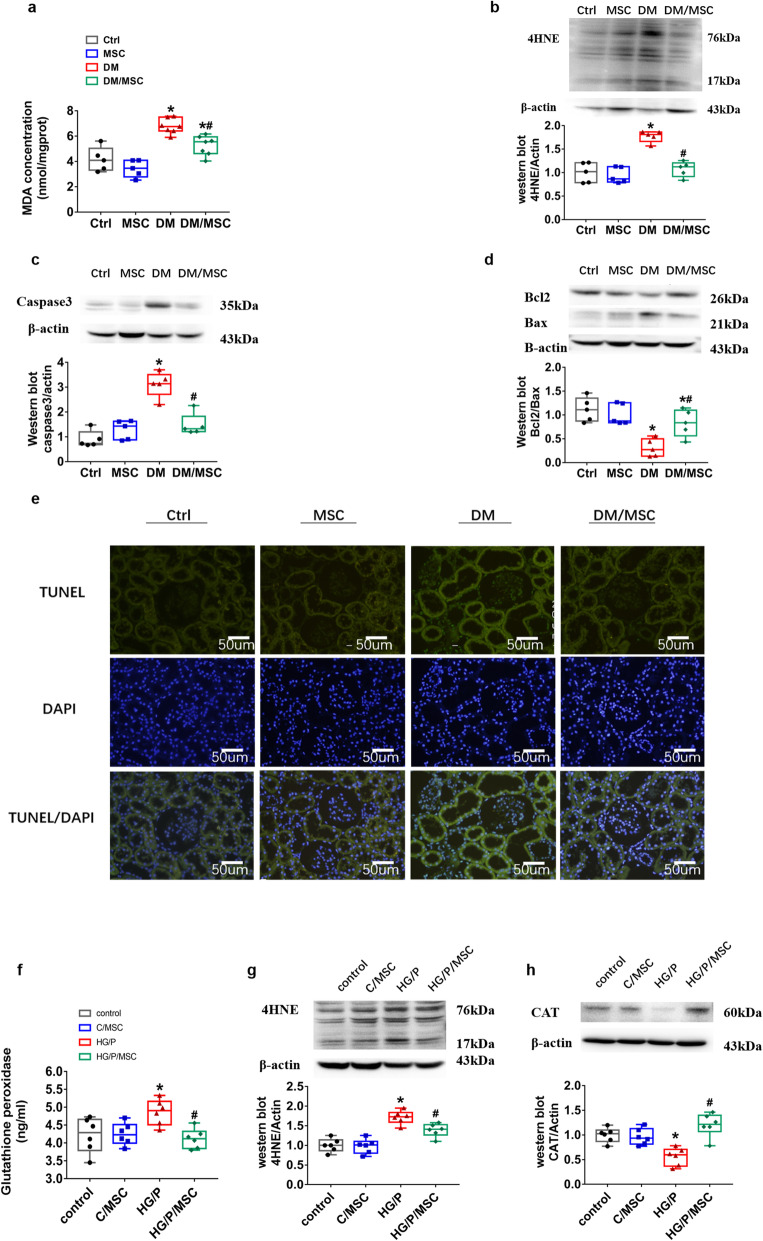
Human umbilical cord mesenchymal stem cells (hUCMSCs) inhibit diabetes-induced oxidative stress and renal cell apoptosis. **a** Level of MDA as a marker of oxidative stress. **b**–**d** Protein level of 4-HNE as a marker of oxidative stress and those of caspase 3, Bcl-2 and Bax as markers of renal apoptosis evaluated via western blotting. **e** TUNEL assay was performed to evaluate apoptosis in renal tissue (green, scale bar = 50 μm). *n* = 5–7 per group. *P < 0.05 vs. Ctrl group; ^*#*^*P <* 0.05 vs DM group. The effects of hUCMSCs on hGMCs were evaluated by measuring the GPX level in the supernatant of cell culture medium (**f**), protein level of 4-HNE (**g**), and CAT (**h**). *control*, control group; C/*MSC*, control and hUCMSC group; *HG/P*, high-glucose and palmitate group; *HG/P/MSC*, high-glucose, palmitate, and hUCMSC group. n = 6 per group. *P < 0.05, vs. control group; ^#^*P* < 0.05, vs. HG/P group

**Fig. 4:**
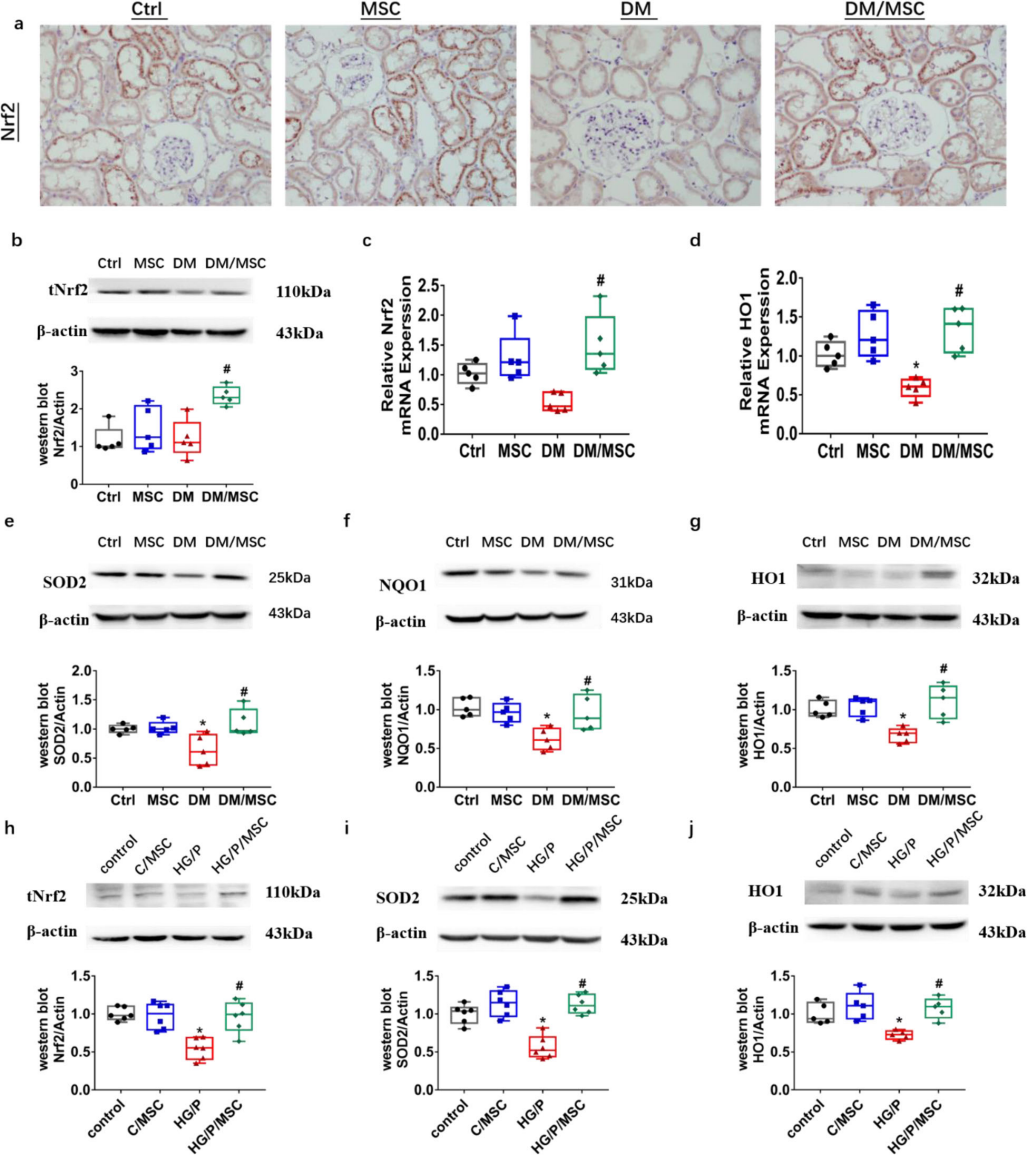
Human umbilical cord mesenchymal stem cells (hUCMSCs) upregulated renal Nrf2 and downstream cytokine expression. **a** Immunohistochemical staining of Nrf2 (× 600). Effects of hUCMSCs on diabetic rats were compared at the protein level for total Nrf2 (**b**), HO-1 (**g**), NQO1 (**f**), and SOD2 (**e**) and at the RNA level for Nrf2 (**c**) and HO-1 (**d**). *n* = 5 per group. *P < 0.05 vs. Ctrl group; ^#^*P* < 0.05 vs DM group. The effects of hUCMSCs on hGMCs were evaluated by measuring the protein levels of total Nrf2 (**h**), SOD2 (**i**), and HO-1 (**j**). *control*, control group; C/*MSC*, control and hUCMSC group; *HG/P*, high-glucose and palmitate group; *HG/P/MSC*, high-glucose, palmitate, and hUCMSC group. *n* = 6 per group. *P < 0.05, vs. control group; ^#^*P* < 0.05, vs. HG/P group

Oxidative stress is important in the development and progression of DN. Therefore, we investigated the presence of malondialdehyde (Fig. 3a) and 4-HNE (Fig. 3b), which are indicators of oxidative damage. We found that malondialdehyde levels in the renal tissue were significantly higher in diabetic rats (DM) than in control rats (Ctrl), which was reversed by hUCMSC treatment (DM/MSC; *P <* 0.05, Fig. 3a). The expression of 4-HNE protein in DM rats was significantly higher than that in the control group but the expression of 4-HNE decreased after hUCMSC treatment (DM/MSC).

In addition, we detected the changes of enzymes related to oxidative damage in vitro. We found that the content of GPX in the supernatant and the protein expression levels of CAT of HG/P hGMCs was significantly lower than that of the control group. The levels of GPX and CAT were significantly increased upon co-culture with hUCMSCs in HG/P/MSC (*P* < 0.05, Fig. 3f, h), while co-culture with hUCMSCs could decrease the expression of 4HNE in hGMCs (*P* < 0.05, Fig. 3g).

A TUNEL assay was performed to analyse apoptosis in the kidney tissue, which revealed more apoptotic cells in DM rats than in control rats; hUCMSC treatment decreased this apoptosis (Fig. 3e). We then conducted western blotting to measure the expression of the apoptosis-related proteins caspase3 and Bax and the anti-apoptotic protein Bcl-2. Caspase3 and Bax levels were significantly increased in DM rats; treatment with hUCMSCs suppressed this increase in DM/MSC rats (Fig. 3c, d, *P <* 0.05). Moreover, the levels of Bcl-2 were decreased in DM rats, which was dramatically reversed by hUCMSC treatment in DM/MSC rats (Fig. 3d, *P <* 0.05).

### Effects of hUCMSCs on Nrf2 expression and its downstream factors

Previous studies have suggested that Nrf2 is involved in regulating antioxidant damage and apoptosis in MSCs; therefore, we measured the protein expression and mRNA levels of Nrf2 by western blotting and qPCR. Total Nrf2 protein and mRNA levels were decreased in DM diabetic rats and increased in DM/MSC rats following hUCMSC treatment (Fig. 4b, c). According to immunohistochemistry staining of the renal tissue, the expression of Nrf2 in both the glomeruli and renal tubules of the DM/MSC group was increased. Measurement of the mRNA and protein expression of downstream factors of Nrf2 by qPCR and western blotting, respectively, showed that the expression of SOD2, HO-1, and NQO1 was decreased in DM diabetic rats compared to that in control rats, all of which were upregulated in DM/MSC rats after hUCMSC treatment (*P* < 0.05, Fig. 4d–g).

Similar results were obtained in vitro. The expression levels of Nrf2 and its downstream factors HO-1 and SOD2 in HG/P hGMCs were significantly lower than those in the control group. The protein expression levels of Nrf2, HO-1, and SOD2 were significantly increased upon co-culture with hUCMSCs in HG/P/MSC (*P* < 0.05, Fig. 4h–j).

### HUCMSCs promote Nrf2 nuclear translocation in vivo and in vitro

**Fig. 5:**
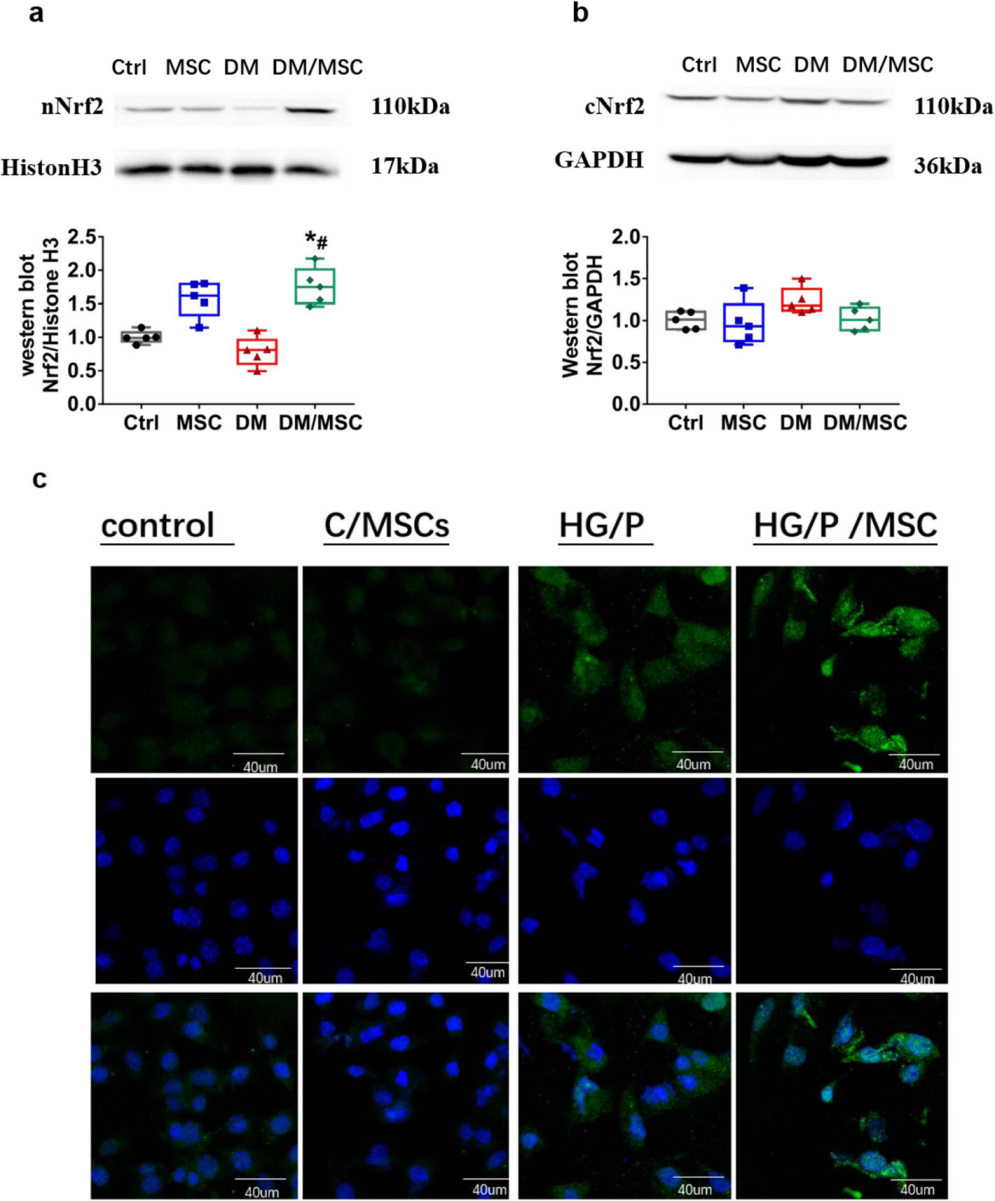
Human umbilical cord mesenchymal stem cells (hUCMSCs) upregulated renal Nrf2 expression. The effects of hUCMSCs on diabetic rats were compared at the protein level for nuclear Nrf2 (**a**) and cytoplasmic Nrf2 (**b**). *n* = 5 per group. **c** Immunofluorescence staining for Nrf2 was performed (scale bar = 40 μm). *P < 0.05 vs. Ctrl group; ^#^*P* < 0.05 vs DM group

When oxidative stress increases, Nrf2 is translocated to the nucleus and promotes the expression of downstream factors. We conducted in vivo and in vitro experiments to verify whether hUCMSCs can promote Nrf2 nuclear translocation in type 2 diabetes. We isolated nucleoprotein from the kidney tissue of diabetic rats and found that after treatment with hUCMSCs, the level of nuclear Nrf2 protein was increased significantly, whereas there was no significant difference in cytoplasmic Nrf2 among groups (Fig. 5a, b).

In addition, we detected Nrf2 expression in hGMCs by immunofluorescence. The expression of Nrf2 was significantly higher after co-culture with hUCMSCs than in the HG/P group. Moreover, Nrf2 expression in the nucleus was significantly increased (*P* < 0.05, Fig. 5c).

### Key role of Nrf2 in hUCMSC-mediated anti-apoptotic activity in vitro

**Fig. 6:**
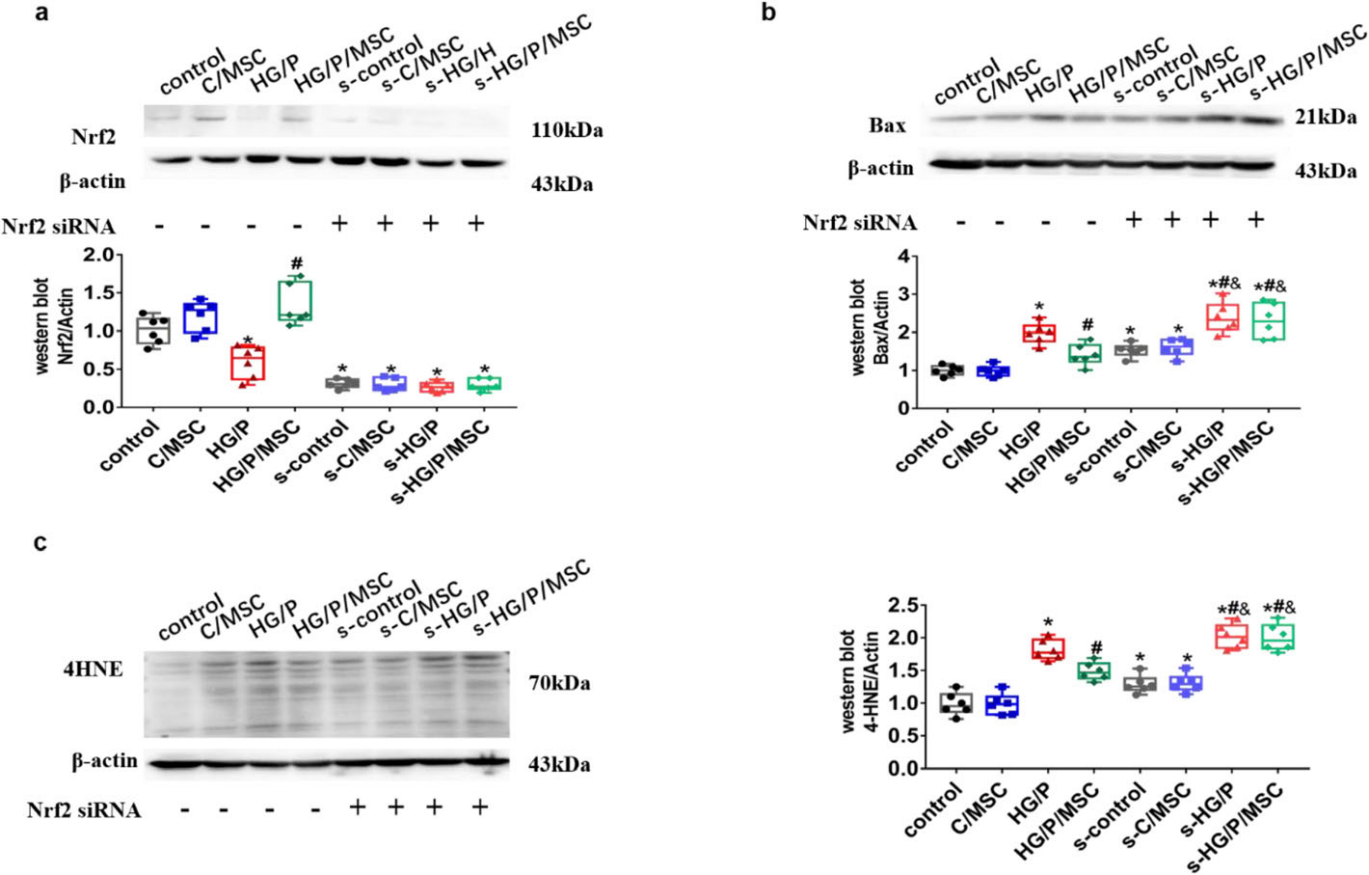
Effect of Nrf2 on oxidative stress and apoptosis in human glomerular mesangial cells (hGMCs) in response to HG/P and human umbilical cord mesenchymal stem cell (hUCMSC) treatment determined by Nrf2 siRNA transfection. Protein expression of **a** Nrf2, **b** Bax, and **c** 4-HNE in hGMCs was measured via western blotting. *control*, control group; C/*MSC*, control and hUCMSC group; *HG/P*, high-glucose and palmitate group; *HG/P/MSC*, high-glucose, palmitate, and hUCMSC group; *s-control*, control group with Nrf2 siRNA; *s-*C/*MSC*, control and hUCMSC group with Nrf2 siRNA; *s-HG/P*, high-glucose and palmitate group with Nrf2 siRNA; *s-HG/P/MSC*, high-glucose, palmitate, and hUCMSC group with Nrf2 siRNA. *n* = 6 per group. *P < 0.05, vs. control group; ^#^*P* < 0.05, vs HG/P group; ^&^*P* < 0.05, vs. Nrf2 siRNA control group

To further demonstrate the key role of Nrf2 in hUCMSC-mediated anti-apoptotic activity in type 2 diabetes, we used RNA interference to inhibit the expression of Nrf2 and evaluated the effect of hUCMSCs on oxidative damage and apoptosis in hGMCs (Fig. 6). After transfection of Nrf2 siRNA, the expression of Nrf2 in hGMCs was very low (Fig. 6a), indicating that Nrf2 transfection was successful. After inhibition of Nrf2, the expression of 4HNE and bax in hGMCs were higher than those in the control group. After co-cultured with hUCMSC, the expression of 4-HNE and Bax in s-HG/P/MSC cells was not significantly decreased compared with that in the s-HG/P group (Fig. 6b, c).

### hUCMSCs induce activation of the PI3K/Akt pathway in hGMCs

**Fig. 7:**
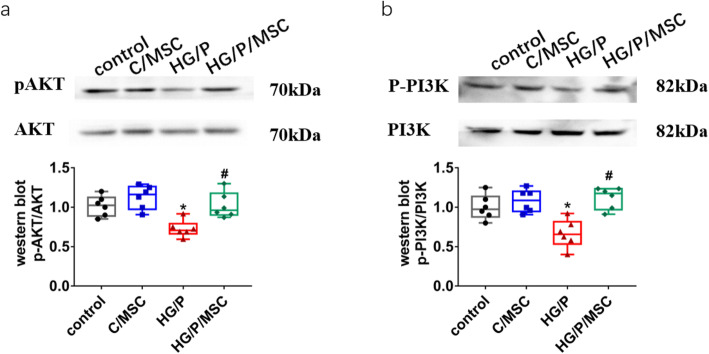
Human umbilical cord mesenchymal stem cells (hUCMSCs) induce activation of the PI3K/Akt signalling pathway in human glomerular mesangial cells (hGMCs). Representative western blots of p-Akt, total Akt (**a**), p-PI3K, and total PI3K (**b**). *n* = 6 per group. *P < 0.05, vs. control group; ^*#*^*P <* 0.05, vs HG/P group

The PI3K/Akt pathway is an important signalling pathway that regulates Nrf2. To confirm whether hUCMSCs regulate Nrf2 through this pathway to improve oxidative damage and apoptosis in DN, we measured phosphorylated Akt and PI3K by western blotting in vitro. As shown in Fig. 7, the levels of phosphorylated Akt and PI3K were downregulated in the HG/P group compared to the control group. In addition, there were significant increases in the phosphorylation of Akt and PI3K in the HG/P/MSC group (*P* < 0.05).

## Discussion

**Fig. 8:**
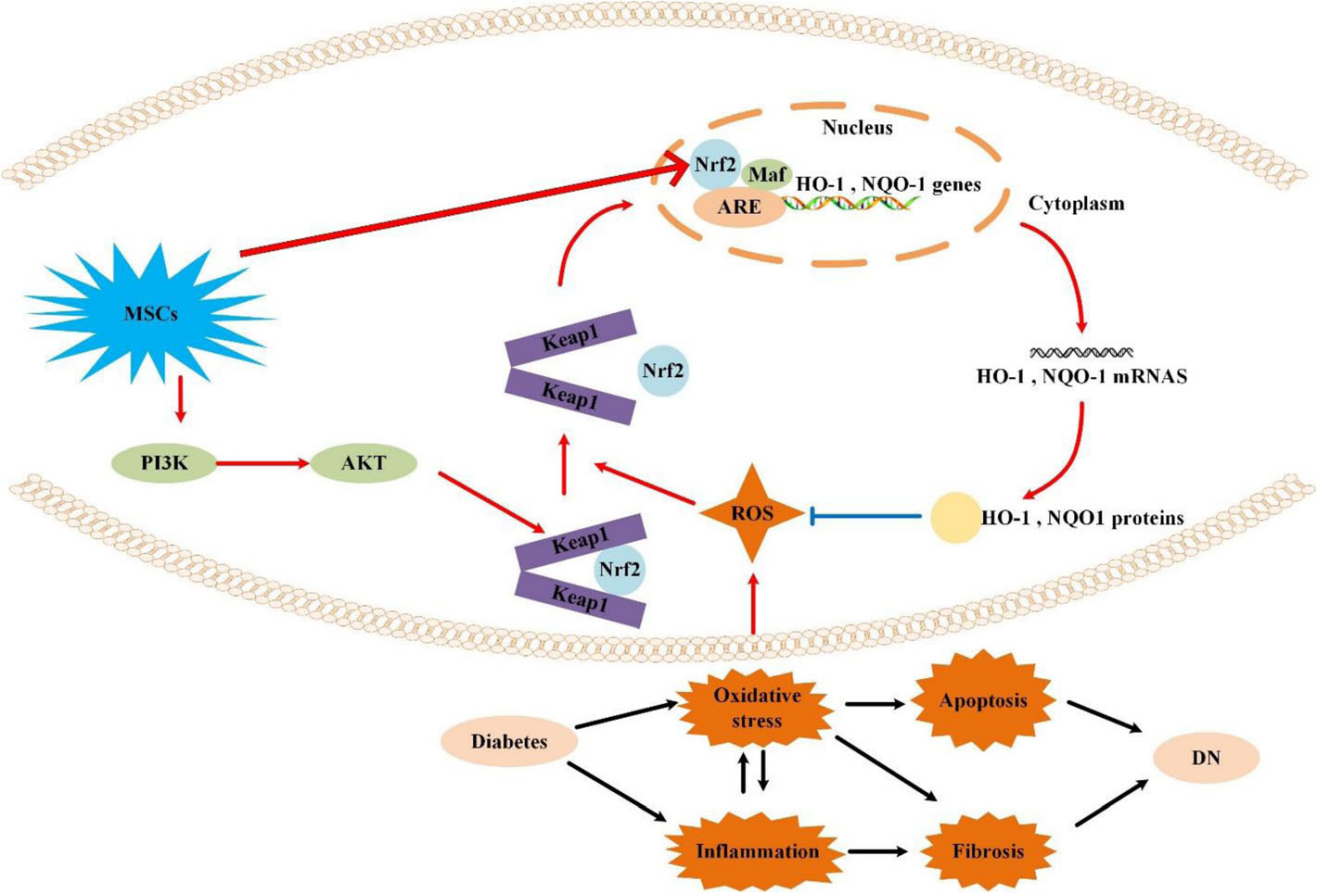
Possible mechanisms for preventing diabetic nephropathy using human umbilical cord mesenchymal stem cells (hUCMSCs)

In this study, we confirmed the protective effect of hUCMSCs on the kidney in type 2 diabetes through in vivo and in vitro experiments. hUCMSCs not only increase Nrf2 expression, but also promote Nrf2 nuclear translocation. We silenced the expression of Nrf2 in hGMCs and found that Nrf2 was a key factor in treatment using hUCMSCs in DN. In addition, hUCMSCs activated the PI3K/Akt signalling pathway upstream of Nrf2 (Fig. 8).

DN is a common complication of type 2 diabetes, which seriously affects the quality of life of patients. MSCs are undifferentiated cells with self-renewal ability, multi-differentiation potential, and low immunogenicity. They can produce a variety of cytokines and growth factors and exert haematopoietic support, immune regulation, and anti-inflammatory functions. Therefore, MSCs may be a new and effective treatment for DN. As a subtype of MSCs, hUCMSCs show good prospects for this application, as these cells are easy to obtain.

Many studies have confirmed that intravenous transplantation of MSCs can significantly reduce blood glucose levels in diabetic animals [18, 19]. Moreover, Wang et al. suggested that transplanted MSCs do not affect blood glucose levels [20]. In the present study, we observed that after three injections, hUCMSCs significantly reduced blood glucose levels in diabetic rats. Additionally, hUCMSCs reduced proteinuria, which agrees with the results of Lv et al. [18]. Although previous studies showed that MSC transplantation causes a decrease in serum creatinine levels, there was no significant difference in the serum creatinine levels between the DM/MSC group and DM group in our study. This may be because diabetic rats were in the early stage of disease, during which changes in renal function are not obvious, preventing detection of the treatment effect. Glomerular hypertrophy and the mesangial matrix were increased significantly, the glomerular basement membrane was thickened, and Kimmelstiel–Wilson nodules and tumour-like dilatation did not occur, indicating that the rats were in the early stage of DN. hUCMSCs can improve these pathological changes in the early stages of DN.

Oxidative damage caused by hyperglycaemia and hyperlipidaemia is among the main causes of DN [21]. Apoptosis of renal parenchymal cells is also an important cause of diabetes-related renal damage [22]. Continuous hyperglycaemia can induce the formation of large amounts of reactive oxygen species [23], which accumulate in renal parenchymal cells and lead to cell apoptosis and DN progression [24]. Malondialdehyde [25] and 4-HNE [26] are important indicators of oxidative and nitrification damage. Both GPX and CAT are important enzymes for anti-oxidation [27]. In this study, hUCMSCs reduced the content of malondialdehyde and expression of 4-HNE protein in the kidneys of diabetic rats and increased the content of CAT and GPX of hGMCs, indicating that hUCMSCs can improve oxidative stress in diabetes. Caspase3 is an important apoptotic protein; an abnormal Bcl-2/Bax ratio, that is, decreased or increased expression of anti- or pro-apoptotic genes, respectively, can lead to excessive apoptosis, resulting in target organ damage. Our study showed that hUCMSCs can improve apoptosis in DN.

HO-1, SOD2, and NQO1 are downstream antioxidant genes of Nrf2 [28, 29]. We investigated whether hUCMSCs can activate Nrf2 and reduce oxidative damage in DN. Our results showed that hUCMSCs increased the expression of Nrf2 and promoted the expression of HO-1, SOD2, and NQO1. We further demonstrated that hUCMSCs promoted the expression of Nrf2 and its downstream factors in a high-glucose and high-fat hGMC model in vitro.

Studies have confirmed that Nrf2 is activated mainly through three pathways: increasing its expression, increasing its nuclear translocation, and reducing its degradation. Under physiological conditions, Keap1 in the cytoplasm binds to Nrf2, inhibits Nrf2 entry into the nucleus, and Nrf2 ubiquitinates and degrades rapidly. Under oxidative stress conditions, reactive oxygen species or electrophilic molecules inhibit the binding of Keap1 and Nrf2 [30]. Nrf2 enters the nucleus and activates the transcription of downstream genes, improving the ability of cells to resist oxidative stress [10]. To determine whether hUCMSCs promote Nrf2 nuclear translocation in addition to increasing Nrf2 expression, we evaluated nuclear protein from the rat kidney tissue to detect the expression of Nrf2 in the nucleus of each group. We found that hUCMSCs significantly increased Nrf2 expression in the nucleus but did not affect the level of Nrf2 in the cytoplasm, suggesting that hUCMSCs not only increase Nrf2 expression, but also promote its translocation. Using a high-glucose and high-fat hGMC model for co-culture with hUCMSCs, we observed that hUCMSCs increased the expression of Nrf2 in hGMC nuclei.

hUCMSCs may play a role in anti-oxidative damage and anti-apoptosis by activating Nrf2 in DN. To confirm whether Nrf2 is the key factor in this process, we silenced Nrf2 expression. The protective effect of Nrf2 on oxidative damage and apoptosis of DN partially disappeared, indicating that Nrf2 plays an important role in anti-oxidative damage and apoptosis in hUCMSCs in diabetes.

Many recent studies have confirmed that PI3K/Akt is the upstream signal molecule of Nrf2/HO-1 [31] and participates in Nrf2 activation [32]. As the binding region of Nrf2 and Keap1 is also the intracellular actin binding site, PI3K promotes the dissociation of Nrf2 and Keap1 and enters the nucleus by altering the arrangement and depolymerisation of actin [31, 33]. Activation of the PI3K/Akt signalling pathway can reduce oxidative stress-induced apoptosis [34]. We found that the expression of phosphorylated Akt and PI3K was significantly decreased in HG/P hGMCs, and hUCMSCs upregulated the expression of phosphorylated Akt and PI3K. hUCMSCs can prevent inactivation of the PI3K/Akt signalling pathway, indicating that hUCMSCs maybe involved in mitigating apoptosis of hGMCs through the PI3K/Akt signalling pathway.

## Conclusions

Collectively, our findings indicate that hUCMSCs can improve apoptosis in DN by promoting antioxidant damage. Nrf2 activation is one of the important mechanisms in this process. These effects may be achieved by activating the PI3K/Akt pathway. Our research also had some limitations, such as that the specific manner by which hUCMSCs affect the PI3K/Akt signalling pathways was not evaluated. In further studies, we will use PI3K inhibitors to examine the mechanism of how hUCMSCs affect the PI3K/Akt pathway.

## Supplementary Information

**Additional file 1:Fig. S1**. Identification of human umbilical cord mesenchymal stem cells. A fat formation (X200); B osteogenesis (X40); C chondrogenesis (X200); D flow cytometry was used to detect the phenotype of human umbilical cord mesenchymal stem cells.

## Data Availability

The datasets and resources generated and analysed during the current study are available from the corresponding author upon reasonable request.

## Abbreviations

DN: Diabetic nephropathy
hUCMSC: Human umbilical cord mesenchymal stem cell
hGMC: Human glomerular mesangial cell
MSC: Mesenchymal stem cell
ND: Normal diet
Nrf2: Nuclear factor erythroid 2-related factor 2
PBS: Phosphate-buffered saline
