# Supplementary material for: Human umbilical cord mesenchymal stem cells reduce oxidative damage and apoptosis in diabetic nephropathy by activating Nrf2
Source: Stem Cell Res Ther. 2021 Aug 11;12:450. doi: 10.1186/s13287-021-02447-x (PMC8356418; doi:10.1186/s13287-021-02447-x)
Supplement: Supplementary file 1 — Additional file 1:Fig. S1. Identification of human umbilical cord mesenchymal stem cells. A fat formation (X200); B osteogenesis (X40); C chondrogenesis (X200); D flow cytometry was used to detect the phenotype of human umbilical cord mesenchymal stem cells. [file 13287_2021_2447_MOESM1_ESM.docx]

Figure S1


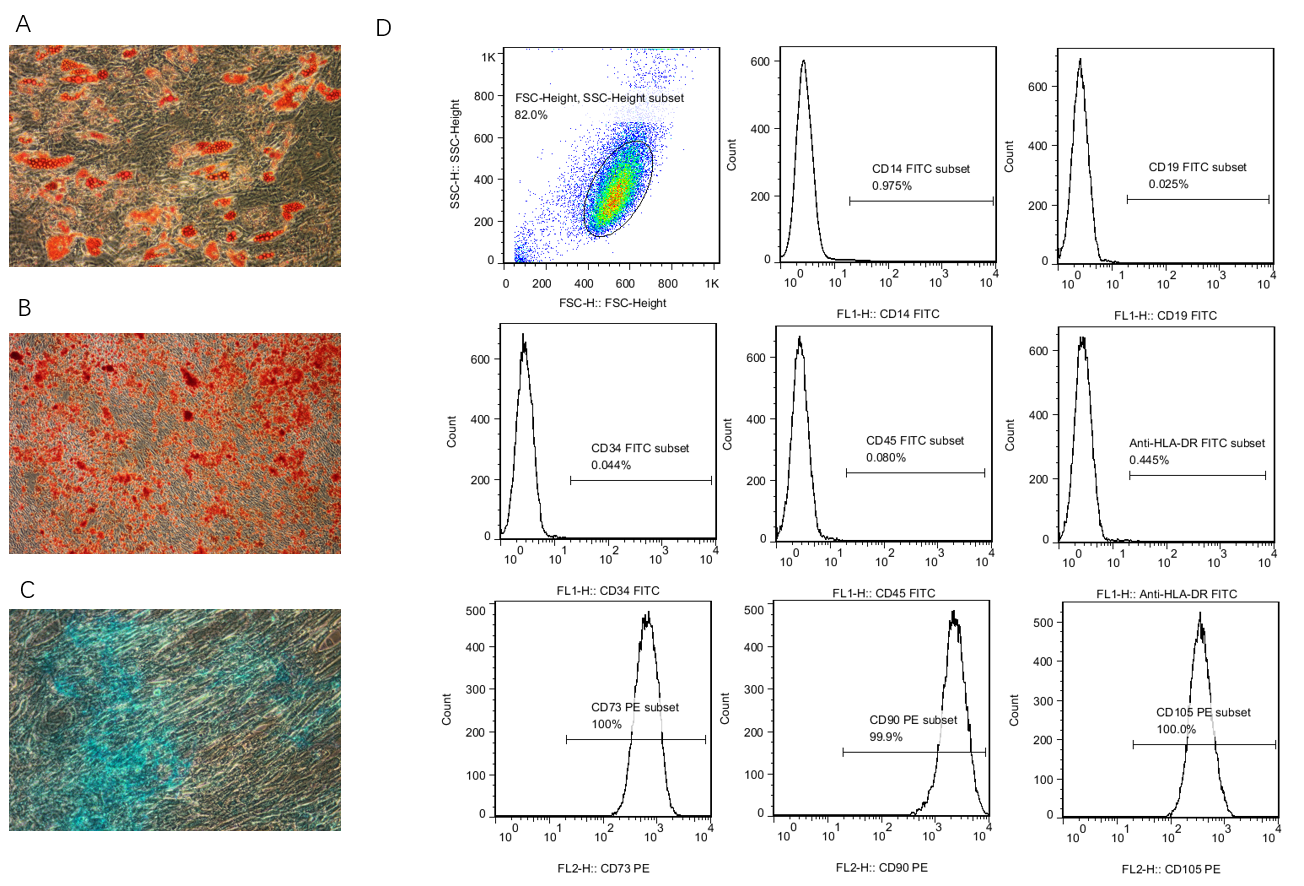


Supplementary materials Figure 1 identification of human umbilical cord mesenchymal stem cells

A fat formation (X200); B osteogenesis (X40); C chondrogenesis (X200); D flow cytometry was used to detect the phenotype of human umbilical cord mesenchymal stem cells.
